# Diagnosing glaucoma

**Published:** 2019-12-17

**Authors:** Madhu Bhadauria

**Affiliations:** 1 Director: CMO, Head of Glaucoma Services, Sitapur Eye Hospital, Sitapur, India.

**Glaucoma is a condition that can lead to severe vision loss if not detected on time. Opportunistic screening is one way to screen for glaucoma.**

**Figure:**
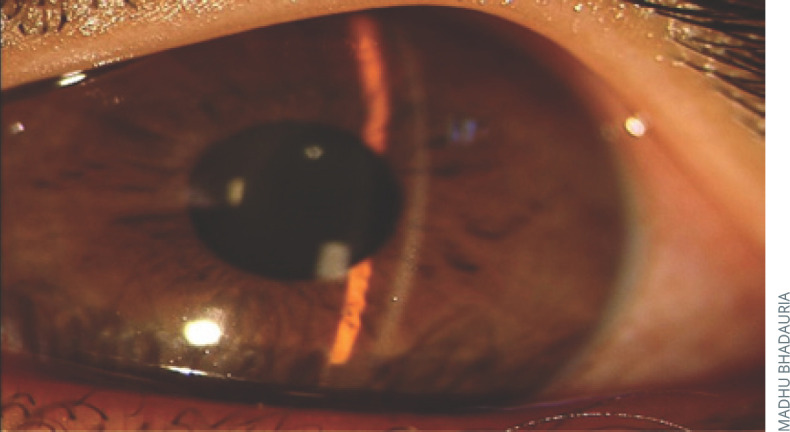
Shallow anterior chamber seen with a torchlight. INDIA

Glaucoma is a disease that damages the eye's optic nerve. It is often a chronic, progressive and degenerative disease that can lead to visual defects. There may also be an acute presentation with redness, pain, tearing and photophobia due to acute raised intraocular pressure (IOP) in cases with angle closure.^1^,^2^ The damage caused by glaucoma is irreversible. This is why it is important to diagnose the disease early to prevent further vision loss.

You can suspect glaucoma in a patient if the patient has:

- family history of the condition
- high refractive errors
- diabetes
- symptoms like coloured halos and/or pain,
- frequent change of glasses
- raised intraocular pressure
- occludable angles
- signs of optic nerve head damage

India is a country of nearly 1380 million people of which about 345 million people (25 per cent) are aged 40 years and above. This age group is eligible for opportunistic screening for glaucoma.

## Opportunistic screening for glaucoma

Opportunistic screening involves checking those at risk for glaucoma when they present themselves for any eye examination. It can be done in outreach camps, vision centres and ophthalmology clinics. Opportunistic screening for glaucoma includes:

- checking for family history of glaucoma
- measuring IOP
- examining the anterior segment with torchlight, including relative afferent pupil defect (RAPD)
- evaluating the optic disc with direct ophthalmoscope

If available it can also include obtaining an image of the optic disc with smart phone photography.

Eye trained staff at outreach camps, vision centres and eye clinics can examine those at risk of glaucoma (anyone aged 40 years and over); this includes patients who may present with presbyopia, refractive errors or cataract. Proper family history, measurement of intraocular pressure, torchlight examination and optic nerve head assessment is recommended for all patients for opportunistic screening of glaucoma.

## Comprehensive ocular examination for glaucoma

### Slit-lamp evaluation for glaucoma

Van Herick technique is used to evaluate anterior chamber depth with a slit-lamp to look for:

- pseudo exfoliation
- neovascularisation of iris
- iris atrophy
- presence of peripheral iridotomy
- blebs
- pigments of corneal endothelium (Kruckenbergh spindle)
- pigments on the anterior surface of the lens

### Tonometry

Do remember to measure IOP of all patients above 40 years at every visit. Applanation tonometer is ideal but rebound or non-contact tonometer can also be used. Corrected IOP according to corneal thickness is useful in suspected cases of ocular hypertension and normal-tension glaucoma.

### Gonioscopy

Gonioscopy is essential for all patients suspected of glaucoma. It examines the angle of the anterior chamber. It is best performed using four-mirror indentation gonioscope. The ophthalmologist should assess the angle as occludable or open as the treatment will depend on the assessment. An angle is occludable when posterior trabecular meshwork is not seen in 180 degrees of angle and more.

Dynamic or manipulative gonioscopy assesses if angle closure is only appositional or if peripheral anterior synechiae are formed. Evidence of blotchy pigments, neovascularisation, excessive pigments on trabecular mesh with wide open angle and concave iris are signs of pigmentary glaucoma.

### Disc evaluation

The best way to evaluate a disc is with a 78 or 90 Dioptre non-contact fundus lens on a slit lamp. It gives a stereoscopic view of the disc to assess optic disc size, cup and rim delineation. In patients suspected of glaucoma important signs to note are: cup size and depth, loss of rim, notches, slopes, and disc haemorrhage. A point to remember is that the margin of cup is where vessels bend and not the area of pallor.

**Figure:**
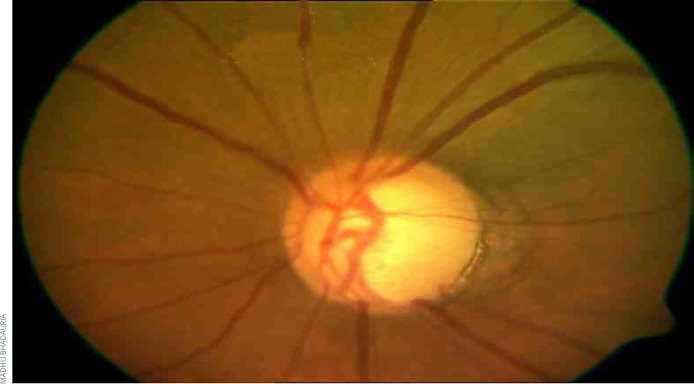
Glaucomatous discs with thinning inferior disc rims. INDIA

The disc suspected for glaucoma may include:

- a vertical cup to disc ratio of more than 0.5
- asymmetric cups (that is a cup disc ratio between both the eyes of more than 0.2)
- notching of the neuro retinal rim and
- splinter haemorrhages at the disc

Disc damage likelihood score (DDLS) is a tool that can help to classify optic discs of different sizes as normal, disc at risk, glaucoma damage and glaucoma disability.

### Visual field analysis

All the patients who are glaucoma suspects need perimetry to check for glaucomatous field loss.

### Imaging in glaucoma

Visual field defects begin to be obvious after a loss of about 40 per cent of retinal ganglion cells. Hence structure imaging tools that can pick up abnormalities earlier have become popular. These tools include optic disc and retinal nerve fibre layer (RNFL) imaging for disc documentation and RNFL loss. The most popular technique is optical coherence tomography (OCT). OCT is a non-invasive test that provides images of disc, RNFL and ganglion cell count of macula. These are useful for early detection and to track progression.

### Progression of glaucoma

Family history, refractive errors, and age are risk factors for progression of glaucoma.^3^ Progression is tracked using IOP, visual fields, disc photos and/or OCT. Optic disc progression can be seen as neuro-retinal rim thinning, enlargement of the cup/ disc ratio and increased area of parapapillary atrophy. Visual field progression is assessed by increasing mean deviation and pattern standard deviation, enlargement of scotoma or increased depth of scotoma. OCT gives numeric values of disc parameters and RNFL thickness; a reduction of ten per cent or more from a previous visit is considered progression. Visual fields and OCT both have built-in progression analysis package called GPA that is capable of giving trend and event analysis.
